# Wnt Signaling in Neural Crest Ontogenesis and Oncogenesis

**DOI:** 10.3390/cells8101173

**Published:** 2019-09-29

**Authors:** Yu Ji, Hongyan Hao, Kurt Reynolds, Moira McMahon, Chengji J. Zhou

**Affiliations:** 1 Department of Biochemistry and Molecular Medicine & Comprehensive Cancer Center, University of California at Davis, School of Medicine, Sacramento, CA 95817, USA; ksreynolds@ucdavis.edu; 2 Institute for Pediatric Regenerative Medicine, UC Davis School of Medicine and Shriners Hospitals for Children, Sacramento, CA 95817, USA; mmcmahon2000@berkeley.edu; 3 Graduate Program of Biochemistry, Molecular, Cellular and Developmental Biology, University of California, Davis, CA 95616, USA; hyhao@ucdavis.edu; 4 Department of Molecular and Cellular Biology, University of California, Davis, CA 95616, USA; 5 College of Letters & Science, University of California, Berkeley, CA 94720, USA

**Keywords:** Wnt, neural crest stem cells, neural crest-derived cancer

## Abstract

Neural crest (NC) cells are a temporary population of multipotent stem cells that generate a diverse array of cell types, including craniofacial bone and cartilage, smooth muscle cells, melanocytes, and peripheral neurons and glia during embryonic development. Defective neural crest development can cause severe and common structural birth defects, such as craniofacial anomalies and congenital heart disease. In the early vertebrate embryos, NC cells emerge from the dorsal edge of the neural tube during neurulation and then migrate extensively throughout the anterior-posterior body axis to generate numerous derivatives. Wnt signaling plays essential roles in embryonic development and cancer. This review summarizes current understanding of Wnt signaling in NC cell induction, delamination, migration, multipotency, and fate determination, as well as in NC-derived cancers.

## 1. Introduction

Neural crest (NC) cells are a unique group of cells originated from the dorsal margins of the neural tube during early vertebrate development [1,2]. They migrate extensively into most tissue/organs and generate numerous differentiated cell types. Consequently, the NC has long been designated the “fourth germ layer” despite originating from the ectoderm. NC development begins during gastrulation, induced at the junction between the neural plate and non-neural ectoderm [3]. After induction, NC cells delaminate from the neuroepithelium by undergoing epithelial-mesenchymal transition (EMT) and migrate long distances in distinct streams according to their positions along the anterior-posterior axis within the embryo [4,5]. Subsequently, NC cells differentiate into various cell types to contribute to different organs in a region-specific manner, including the peripheral nervous system, melanocytes, the adrenal medulla, smooth muscle, as well as various skeletal, connective, adipose, and endocrine cell subtypes [6,7].

**Figure 1:**
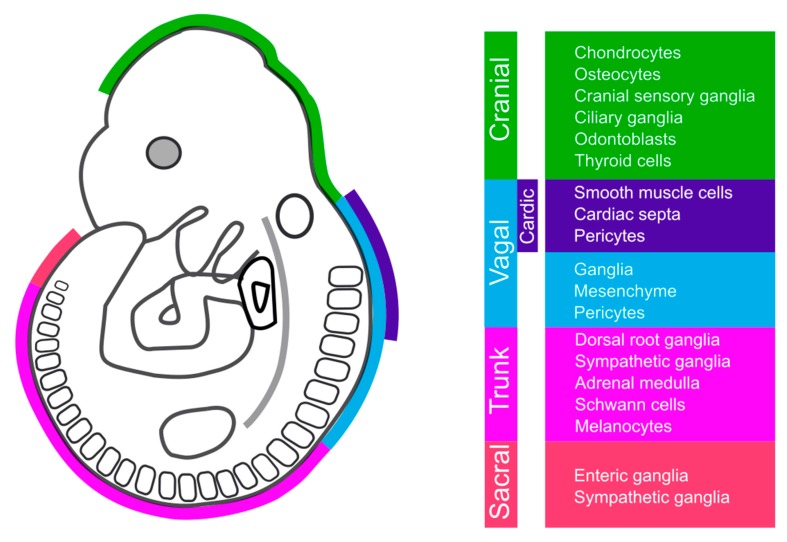
Anatomically distinct neural crest cell populations and their major derivates. Schematic lateral view of a mouse embryo at embryonic day 9.5 shows the cranial (*green*), vagal (*azure*), trunk (*purple*), and sacral (*ruby*) neural crest cells and their major derivatives. Vagal segment includes cardiac neural crest (*indigo*).

NC cells originate from four major segments of the neural tube to form cranial (or cephalic), vagal, trunk, and sacral neural crest cells [8] (Figure 1). Distinct populations of cranial NC cells originate from the diencephalon, midbrain, and hindbrain [9] and have the capability to differentiate into craniofacial bone, cartilage, and connective tissue [10]. The rostral cranial NC cells generate the frontonasal skeleton, while the posterior cranial NC cells fulfill the pharyngeal arches in vertebrates (also known as branchial (gill) arches in fish and amphibians), to generate middle ear, bone and cartilage of the jaw and neck [11]. Cranial NC cells also make essential contributions to the membranous bones of the skull vault [11]. The vagal NC, which arises from somites 1–7, has been depicted as a crossbreed between the cranial and trunk NC [12]. The vagal NC forms various cell types in thymus, thyroid, parathyroid, and heart; and also forms ganglia in lung, pancreas, and the gut [13]. Cardiac NC cells arise from the dorsal neural tube between the otic vesicle and the third somite in the vagal crest segment [13,14]. These cells are required in avians to trigger restructuring of the developing cardiac outflow tract [15]. In mammals, NC-derived cells occupy conotruncal cushions and the aorticopulmonary septum during overt septation of the outflow tract and envelop both the thymus and thyroid as these organs form [16]. Trunk NC cells arise in the posterior part of the embryo and migrate along three distinct pathways: a dorsolateral pathway between the ectoderm and the somites, a ventro-lateral pathway between somites, and a ventro-medial pathway between the neural tube and the posterior sclerotome [17]. These cells generate pigment cells of the skin, the peripheral nervous system, and secretory cells of the endocrine system [18].

Environmental cues or cell-autonomous factors can affect appropriate NC cell differentiation, causing cell cycle dysregulation and ectopic tissue formation [19]. Defects in NC cell development are related with numerous serious diseases, many of which mainly affect children [19]. These abnormalities, named neurocristopathies [20], are one of the commonest birth defects in newborns, including congenital heart defects, craniofacial malformations, and familial dysautonomia [19,21,22]. Hirschsprung disease is caused by the failing of vagal NC cell migration to the colon, leading to the absence of enteric ganglia required for peristaltic bowel movement [23,24]. Treacher Collins syndrome is an uncommon human congenital disorder which presents with craniofacial abnormalities due to excessive apoptosis within a pool of cranial NC cells which migrate to the first and second branchial arches [21]. DiGeorge syndrome is a congenital disease which is also caused by abnormal migration of NC cells into the pharyngeal arches. DiGeorge patients usually present with immunodeficiency, cardiac defects, learning disabilities, psychiatric impairment, and craniofacial malformations [25]. Waardenburg syndrome is an autosomal dominant disorder that often includes hypopigmented patches of skin. In many cases, patients have mutations in *PAX3*, *SNAI2*, or *SOX10*, which are all involved in NC induction and specification [2]. CHARGE syndrome is induced by mutations in helicase DNA-binding protein 7 (*CHD7*) gene, which is critical for maintenance of NC multipotency and migration [26,27]. Patients with CHARGE syndrome have malformation in NC-derived tissues, such as heart defects and coloboma [28].

Niche signals acting prior to and after migration of NC cells are known to be involved in NC development [8]. Strong evidence shows that Wingless-type MMTV integration site family member (Wnt) signaling is involved in various stages of NC development [29,30,31]. Exogenous Wnt activation is sufficient to induce human NC cells from pluripotent stem cells [32]. Wnt signaling is both necessary and sufficient for inducing NC cells in *Xenopus*, chicks, and mice [33,34,35,36]. Furthermore, loss- and gain-of-function experiments reveal that non-canonical Wnt signaling plays an essential role in NC cell migration by regulating actin polymerization [37,38]. In contrast, canonical Wnt signaling may control NC cell lineage differentiation [37,39]. In mice, conditional knockout of intracellular Wnt signal transducer β-catenin (Ctnnb1) in pre-migratory NC cells suppresses both sensory neuron and melanocyte formation [40]. In this review, we summarize current understanding of Wnt signaling in NC development based on published data from the mouse, chick, quail, frog, and zebrafish models, and in NC-derived cancers.

## 2. Wnt Signaling Pathways

### 2.1. β-Catenin-Dependent Canonical Wnt Signaling Pathway

Canonical Wnt/β-catenin signaling plays vital roles in development and disease [41]. In this pathway, Wnt proteins bind to two distinctly different types of cell surface receptors, Frizzled (Fzd) and low-density lipoprotein (LDL) receptor-related protein 6 (Lrp6) or Lrp5 [41]. Fzd family proteins are seven-transmembrane proteins with an extracellular cysteine-rich domain (CRD) [42], which interacts with both the N-terminal domains (D1) and C-terminal domains (D2) of Wnt proteins [43,44]. Lrp5 and Lrp6 are single-span transmembrane co-receptors that bind to the D2 domain of Wnt proteins [44,45,46,47]. Therefore, the canonical Wnt proteins have the ability to bridge these different types of receptors Fzd and Lrp5/Lrp6 [44,45]. The binding of Wnt proteins causes a conformational change of the receptor complex, resulting in phosphorylation of the cytoplasmic domain of Lrp5/Lrp6 by several kinases, such as glycogen synthase kinase 3 (GSK3), allowing the recruitment of the scaffold protein Axin [41]. This leads to the inhibition or saturation of β-catenin degradation [48]. Therefore, the newly synthesized β-catenin can be accumulated and translocated to the nucleus [49,50]. Within the nucleus, β-catenin binds to a T-cell factor/lymphoid enhancer factor (TCF/LEF) transcription factor family member to regulate the expression of genes involved in cell proliferation, differentiation, and apoptosis [51]. Without Wnts, the cytoplasmic β-catenin is continually degraded by the destruction complex [48]. In this complex, Axin acts as a scaffold and interacts with β-catenin, the tumor suppressor protein adenomatous polyposis coli (APC), and two constitutively active kinases, GSK3, and Casein kinase 1 (CK1) [52]. There, β-catenin is phosphorylated by GSK3, causing its ubiquitination and degradation [41].

### 2.2. β-Catenin-Independent Non-Canonical Wnt Signaling Pathways

Among the β-catenin-independent non-canonical Wnt signaling pathways is planar cell polarity (PCP) signaling, which provides positional information to mediate asymmetric cytoskeletal organization [53]. Therefore, Wnt/PCP is essential for tissue patterning and morphogenesis as well as polarized cell migration [53,54]. Binding of Wnt proteins to Fzd receptors can activate and recruit Dishevelled (Dvl or Dsh) to the cell membrane where it forms a complex with Dvl-associated activator of morphogenesis 1 (Daam1) [55]. This complex activates Rho GTPases which leads to the subsequent activation of Rho-associated kinase (Rock) [56]. This contributes to asymmetric cytoskeletal organization and polarized cell migration [57]. Another type of non-canonical Wnt signaling is the Wnt-cGMP/Ca^2+^ signaling pathway, which regulates intracellular Ca^2+^ flux and levels [58]. In this pathway, the binding of Wnt proteins to Fzd leads to the production of inositol 1,4,5-triphosphate (IP3) and diacylglycerol (DAG) [58,59]. IP3 leads to the releasing of Ca^2+^ from the endoplasmic reticulum (ER). DAG is activated by high concentration of calcium released from the ER, which activates protein kinase C (PKC) [60]. Ca^2+^ also activates calcium/calmodulin-dependent protein kinase II (CaMKII) [59]. Both CaMKII and PKC activate various transcription factors, such as NFκB [61].

## 3. Wnt Signaling in NPB Formation and NC Induction

**Figure 2:**
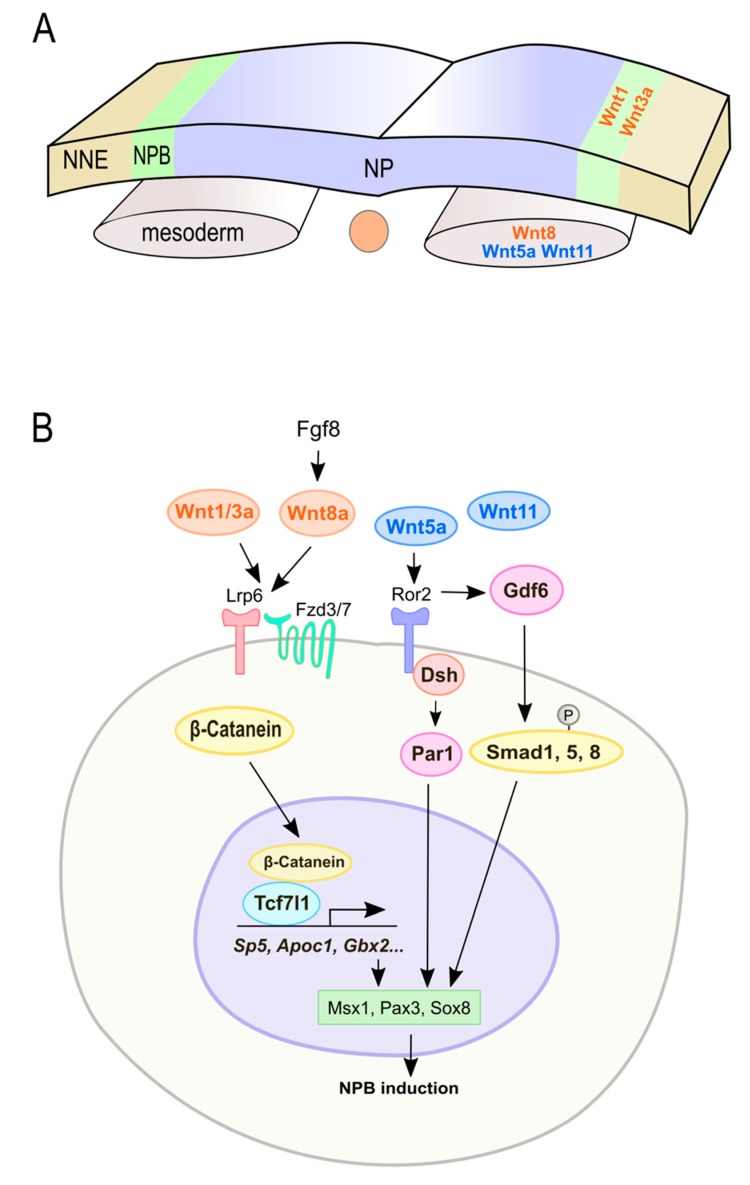
Wnt signaling regulates neural plate border (NPB) induction by regulating NPB specifiers. (**A**) NPB induction begins during gastrulation and is regulated by both canonical (*orange*) and non-canonical (*blue*) Wnts secreted from NPB and paraxial mesoderm. (**B**) Key components and possible interactions between Wnt signaling and NPB specifiers. NNE, non-neural ectoderm; NP, neural plate.

**Figure 3:**
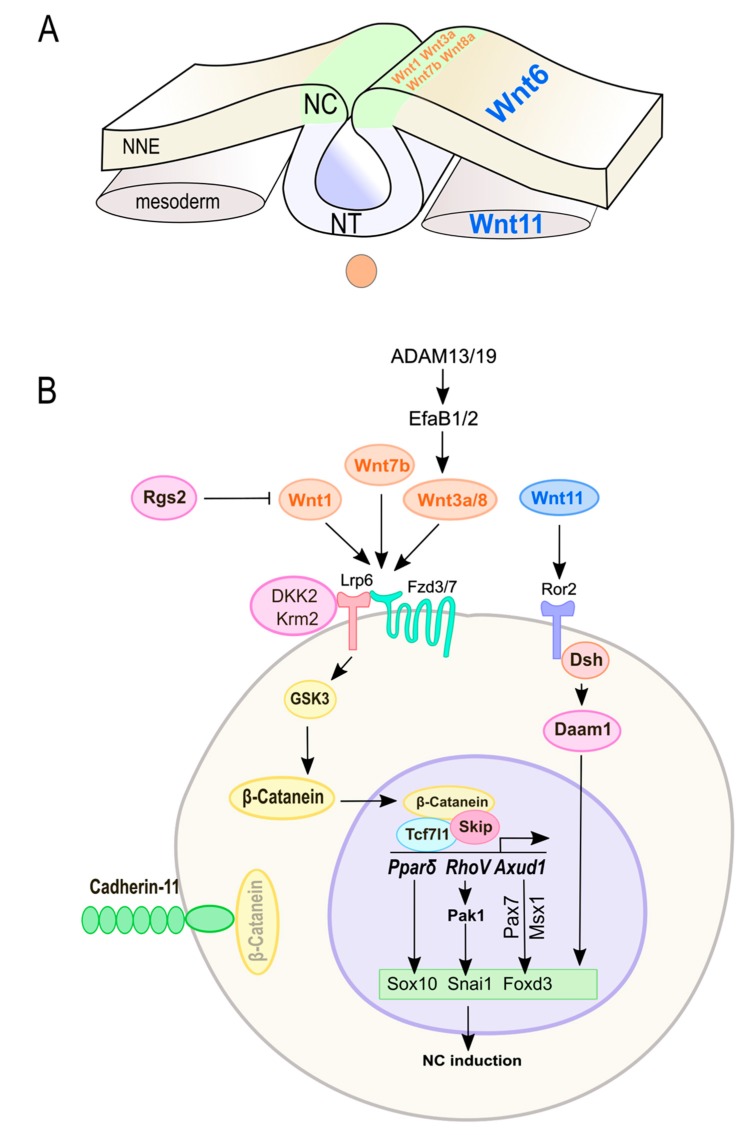
Wnt signaling induces neural crest cells by regulating NC specifiers. (**A**) NC is induced from the neural plate border at the end of gastrulation (zebrafish and *Xenopus*) or the beginning of neurulation (chick and mouse). This process is regulated by both canonical (*orange*) and non-canonical (*blue*) Wnts secreted from NPB, adjacent NNE, and paraxial mesoderm. (**B**) Key components and possible interactions between Wnt signaling and NC specifiers. NC, neural crest; NNE, non-neural ectoderm; NT, neural tube.

NC induction is a multistep process regulated by a complex gene regulatory network [62]. It is initiated during gastrulation and continues through neural tube closure [63]. The vertebrate ectoderm can be separated into three major subdivisions at the end of gastrulation: the non-neural ectoderm, the neural plate, and the neural plate border (NPB) [64] (Figure 2). The non-neural ectoderm will develop into the epidermis, and the neural plate will give rise to the central nervous system [65]. In most experimental vertebrate species, the NPB begets the NC as well as the pre-placodal ectoderm [66]. The induction of NC can be distinguished into three steps. First, inductive signals drive the expression of a set of transcription factors that define the NPB, known as NPB specifiers. Subsequently, the NPB specifiers and inductive signals work together to stimulate another set of transcription factors more restricted to the NC, which are known as NC specifiers [62,63,66]. Finally, inhibitory interactions between the neural plate, the non-neural ectoderm, the anterior neural fold, and the NC acutely define the boundaries between these territories [35,67,68]. NPB specifiers, such as *Zic1*, *Gbx2*, *Msx1*, *Pax3/7*, *Gata2/3*, *Foxi1/2*, *Dlx5/6*, and *Hairy2*, are expressed in the early gastrula [62,63,64,66,69,70,71,72]. NC specifiers include *Snai2*, *Foxd3*, *Sox8/9/10*, *Ets1*, *cMyc*, and *Twist* [62,71,73,74]. Multiple signals regulate the expression of NPB and NC specifiers, including Wnt, Bmp, Fgf, and Notch [62]. Since the formation of NC occurs at the border between the neural plate and non-neural ectoderm, and ventrally adjacent to mesoderm, these tissues have been suggested as the source of NC induction signals [3]. Here, we summarize the involvement of Wnt signaling (in the order of canonical then non-canonical for classified Wnt signaling molecules) in NPB formation and NC induction ( Figure 2 and Figure 3; Table 1). The general description is based on findings on multiple vertebrates, and the species/region-specific findings are clearly noted in the tables and as much as in the text.

### 3.1. Wnt Signaling in NPB Formation

The canonical ligand-encoding genes *Wnt1* and *Wnt3a* are expressed in the NPB (Figure 2A) and dorsal neural tube of mice [104]. In the absence of both *Wnt1* and *Wnt3a*, NPB formation is disrupted, resulting in a defect in neural crest derivatives [101]. Wnt3a expression is modulated by retinoic acid (RA) signaling in mice [77]. Ablation of mouse Raldh2 (Aldh1a2), a RA biosynthetic enzyme [105], delays Wnt3a expression in the dorsal neural tube and decreases the expression of NPB specifiers, such as Msx1 and Pax3 [77]. Unlike *Wnt1* and *Wnt3a*, *Wnt8* is expressed in the paraxial mesoderm (Figure 2A). In *Xenopus*, Fgf8 induces the expression of *Wnt8* in the paraxial mesoderm and regulates the formation of NPB [83] (Figure 2B). Tcf7l1 is a transcription factor activated by Wnt/β-catenin signaling. Inhibiting Tcf7l1’s ability to bind with β-catenin blocks NPB formation (*Msx1*) and NC induction (*Snai2*, *Sox9*) in *Xenopus* [98]. The transcription factor *Gbx2* is a direct target of Wnt/β-catenin signaling, and impaired Tcf7l1 function negatively affects *Gbx2* activation in *Xenopus* [87]. During early NC formation, Gbx2 interacts with the neural fold gene *Zic1* and upregulates the expression of NPB specifiers *Pax3* and *Msx1*. In the absence of Gbx2, Zic1 drives the expression of pre-placodal genes, but Gbx2 activation inhibits pre-placodal fate and induces NC cells [87].

In *Xenopus*, the canonical Wnt/β-catenin signaling could induce the expression of *Apoc1* that belongs to the apolipoprotein family and binds lipids to form lipoprotein particles and function in lipid transport [106]. Depletion of Apoc1 protein resulted in defective formation of a neural plate border (*Msx1*, *Pax3*, *Zic1*) and loss of neural crest cells (*c-Myc*, *Sox9*, *Snai2*, *Twist1*, *Id3*) [78]. The transcription factor *Sp5* has also been shown to be a direct target of Wnt signaling in mice [107]. In *Xenopus*, *Sp5* is induced by Fgf8a or Wnt8 signals to promote NC formation [97]. Sp5 has been shown to regulate the expression of NPB specifiers *Msx1* and *Pax3* and alters *Zic1* expression to promote NC fate during gastrulation [97]. Awp1 is a lipid-activated kinase which associates with the serine/threonine polarity kinase Par1 [108]. In *Xenopus*, Awp1 mediates NPB formation (*Msx1*, *Pax3*) and NC induction (*Sox10*, *Snai2*) by modulating the stability of β-catenin and regulating Wnt signaling [79].

Non-canonical Wnt signaling also plays an important role in NPB formation mainly based on findings in *Xenopus*. Gain- and loss-of-function experiments showed that Wnt5a and Wnt11 are required for the formation of NPB (*Pax3*, *Sox8*) and NC (*Foxd3*, *Snail2*, *Twist*) through Dvl and Ror2 in *Xenopus* [84]. Par1 plays an important role in neural crest induction (*Sox8*, *Foxd3,* and *Snai2*). Wnt5/Wnt11 signaling induce the dissociation of Par1 from the cell cortex, upregulating its enzymatic activity, which then regulates the expression of *Pax3* [84]. Ror2 is a major regulator of non-canonical Wnt signaling [109]. In *Xenopus*, Wnt5a-Ror2 signaling upregulates *Papc* or *Pcns* which then upregulates Bmp ligand *Gdf6,* thus activating Bmp signaling (pSmad1/5/8) in the dorsolateral marginal zone [95]. Moreover, Ror2 regulates cell polarity in the neuroectoderm and shapes the NPB during early neurula stages. *Ror2* loss-of-function causes reduced expression of neural plate border specifiers (*Gbx2*, *Msx1*, *Msx2, Zic1*) and neural crest marker genes (*Twist*, *Snail2*, c-*Myc*, *Tfap2a*) [95].

### 3.2. Wnt Signaling in NC Induction and Specification

Canonical Wnt signaling is well-known to regulate NC induction, and Wnt1, Wnt3a, Wnt7b, Wnt8, Fzd3, and Lrp6 have all been shown to be required for vertebrate NC specification [47,85,88,102,103,110] (Figure 3). In *Xenopus*, the Wnt receptor Fzd7 is required to induce NC markers (*Sox10*, *Sox9*, *Snail*, *Twist*, *Foxd3*). Fzd7 can induce neural crest through binding with different Wnts, including Wnt1, Wnt7b, and Wnt8. Fzd7 may activate both canonical and non-canonical Wnt signaling pathways. *Fzd7* knockdown can be rescued by overexpressing β-catenin, suggesting that Fzd7 regulates neural crest specification through the canonical Wnt pathway in *Xenopus* [86]. Canonical Wnt signaling via Wnt1 regulates the expression of *RhoV* in NC cells, which is required for the induction of NC in *Xenopus* [94]. RhoV has been shown to regulate Pak1 [111], which can phosphorylate and activate Snai1 [112]. Therefore, RhoV may act as mediator of canonical Wnt signaling in NC development [113]. Axud1 is a transcription factor which acts downstream of Wnt/β-catenin signaling during NC induction in chicks [114]. *Axud1* knockdown inhibits the expression of NC specifiers (*Sox9*, *Sox10*, and *Ets1*), but not the NPB gene *Pax7* [80]. Axud1 directly interacts with Pax7 and Msx1 to form a transcriptional complex. This complex can bind to the *Foxd3* NC1 enhancer to regulate *Foxd3* expression [80].

The canonical Wnt mediator β-catenin affects NC survival in mice. Knockout of β-catenin by a Wnt1-driven Cre recombinase causes increased apoptosis in pre-migratory NC cells, suggesting canonical Wnt signaling is needed for the expansion of NC progenitors in mice [115]. However, Wnt1-Cre-mediated reporter activity is first detected about 0.5–1 days after NC induction begins [116]. Therefore, these results may not be able to reject the role of canonical Wnt signaling in NC induction in mice.

Dickkopf-related protein 2 (Dkk2) acts as either an antagonist or activator of Wnt signaling by binding to the Wnt co-receptor Lrp6 [117,118,119]. A recent study in *Xenopus* shows that Dkk2 is required for neural crest induction [34]. Blocking *Dkk2* does not affect the expression of NPB specifiers (*Pax3*, *Sox8*, and *Snai1*) but blocks the formation of neural crest cells by causing a reduction of neural crest specifiers (*Snai2*, *Twist1*, and *Sox10*) [34]. In *Dkk2*-depleted embryos, overexpression of Lrp6 or β-catenin could rescue neural crest formation; however, inhibition of GSK-3β could not, indicating that Dkk2 also activates Wnt/β-catenin signaling independently of GSK-3β [34]. Moreover, *Xenopus* Wnt8 induces NC genes in animal cap explants. *Dkk2* knockdown significantly blocks the induction of *Snail2* by Wnt8, implying two independent mechanisms by either Wnt8 or Dkk2 to activate β-catenin and induce NC formation [34].

The canonical Wnt signaling is also known to be required for anterior-posterior patterning. It has been proposed that NC specification by Wnts is an indirect effect of posteriorization activity rather than a direct effect [120,121]. In *Xenopus*, canonical Wnt signaling induces posterior neural tissue through activation of Fgf signaling [122]. Therefore, the posteriorizing activity of Wnt signaling can be blocked by inhibiting Fgf signaling [82]. In contrast, studies in *Xenopus* show that Wnt proteins could induce NC specifiers (*Snai2*, *Twist*) even if its posteriorizing activity is inhibited, suggesting that NC induction is a direct consequence of Wnt signaling [82].

Although a requirement for canonical Wnt signaling in NC induction has been thoroughly demonstrated, the role of non-canonical Wnt signaling in this process remains understudied. In chicks, *Wnt6* is expressed in the ectoderm and controls the induction of NC through Dvl-mediated non-canonical Wnt signaling [99,100]. Surprisingly, Schmidt et al. showed that canonical Wnt signaling (Wnt1) inhibited neural crest formation in the chicken embryo [100]. These results may indicate evolutionary changes in NC induction during vertebrate evolution. The noncanonical Wnt11 pathway activates the formin family protein Daam1 during NC induction in *Xenopus* [38]. The ability of Daam1 to induce NC formation requires its FH2 domain, which binds to G-actin, suggesting that Daam1 induces NC through actin polymerization in *Xenopus* [38]. However, the role of actin remodeling in NC specification has yet to be defined.

### 3.3. Crosstalk of Wnt Signaling with Upstream Modulators in NC Induction

Many signals control NC induction by regulating canonical Wnt signaling. In zebrafish, regulator of G protein signaling 2 (Rgs2) has been shown to negatively regulate Wnt signaling (*Wnt1*, *Wnt8a*) [93]. Disruption of *Rgs2* expression in zebrafish caused upregulation of many Wnt target genes (*Nfatc2a/b*, *Nfatc3a/b*, *Nfatc4*, *Tcf7l2*, *Axin2*, *Tcf7*, *Pparδ (Pparda/b)*, *Ccnd1*, *Myca/b*, and *Tp53*) and induced neural crest specifiers (*Sox10*, *snail1b*) [93]. Among these genes, the expression of *Pparda* was upregulated at the neural crest progenitor stage. *Pparδ* could bind to the promoter of *Sox10* directly [93]. Therefore, Rgs2-Wnt1/8a–Pparδ–Sox10 signaling mediates neural crest development in zebrafish [93].

Adams are multi-domain transmembrane proteins involved in many developmental processes. Studies have shown that knockdown of two paralogous disintegrin proteases, *Adam13* and *Adam19* in *Xenopus* embryos, inhibits Wnt signaling and the expression of NC specifiers (*Snai2*, *Sox9*, *Foxd3*), but does not affect NPB specifiers (*Pax3*, *Zic1*, *Msx1*) [76]. Moreover, ephrin B1(EfnB1) and ephrin B2 (EfnB2) have been determined as substrates for Adam13 [75]. EfnB1 and EfnB2 are cell-surface ligands for EphB receptor tyrosine kinases which act as antagonists of Wnt signaling [75,123]. Through cleaving EphB2, Adam13 permits Wnt signaling and induces NC cell formation (*Snai2*) [75]. Unlike Adam13, the ability of Adam19 to induce NC specification does not depend on its protease activity [76]. Using immunocytochemistry and immunoprecipitation, the authors further show that Adam19 interacts with Adam13 in the ER and protects Adam13 from ubiquitin-proteasome-mediated degradation in *Xenopus* [75,76].

Cadherin-11 (Cdh11) binds β-catenin at cell-cell adhesion complexes. During NC induction in *Xenopus*, Cdh11 competes with Wnts for the cytoplasmic β-catenin [81]. Depletion of cadherin-11 results in increased levels of β-catenin in the nucleus, and therefore activates canonical Wnt signaling and increases NC marker gene expression (*Sox10*, *AP2*) [81,124].

Kremen 2 (Krm2) is a transmembrane receptor for Wnt antagonists Dkk proteins, and its expression is regulated by canonical Wnts (Wnt3a and Wnt8) in *Xenopus* [125]. Krm2 is required for NC induction in *Xenopus*, and this function is independent from that of Dkk. Krm2 regulates NC induction by direct binding to Lrp6 to promote Wnt signaling in *Xenopus* [90].

Rap2 belongs to the Ras GTPase family. Studies in *Xenopus* showed that Rap2 physically binds with Lrp6 and stabilizes it from proteasome and/or lysosome-dependent degradation [91]. TRAF2/Nck-interacting kinase (TNIK) is a downstream effector of Rap2 that controls the stability of Lrp6 [91]. Rap2/TNIK kinase pathway plays a critical role in Wnt signaling (Wnt8)-mediated NC induction in *Xenopus* [91]. Depletion of *Rap2* could inhibit NC formation (*Snai1, Snai2*, and *Foxd3*) [91].

Skip is a transcriptional co-regulator that plays an important role in NC induction in *Xenopus* [126]. Both under- and overexpression of Skip inhibits Wnt/β-catenin signaling, therefore blocking NC induction (*Snai2*, *Sox3*, and *engrailed2*) in *Xenopus* [126]. Upon overexpression, Skip forms a complex with Lef1 and Hdac1 to repress Wnt target gene expression [126]. However, Skip also interacts with β-catenin and acts as a scaffold in β-catenin/TCF-mediated transcriptional regulation [126]. These results suggest that *Skip* expression level needs to be properly modified during NC induction in *Xenopus* [96].

Finally, inhibiting Wnt signaling in surrounding tissues can sharpen the boundaries between NC and their neighboring territories. In zebrafish, the potassium channel tetramerization domain containing 15 (Kctd15) inhibits NC induction (*Sox10*, *Foxd3*, *Dlx3b*, *Sox9b*, *Tfap2a*, *Snai1b*) by antagonizing Wnt3a signaling and inhibiting the transcription factor Tfap2a, thereby restricting lateral expansion of the neural crest beyond its domain [89,127,128,129]. Hes3 is a member of the Hes family of basic helix-loop-helix transcriptional repressors and is expressed at the boundary of the neural plate [35]. Overexpression of *Hes3* blocks *Snai2* and *Sox10* induction by Wnt8 in *Xenopus*, suggesting that Hes3 establishes the NP/NC boundary by blocking the mesoderm-derived Wnt signals [35]. Tcf7l1 is a transcriptional repressor of canonical Wnt target genes [130]. In mice, *Tcf7l1* is expressed in the anterior neural fold region during neurulation and is required for forebrain development [67]. Conditional inactivation of *Tcf7l1* using AP2α-Cre results in anterior expansion of NC cells, suggesting Tcf7l1 defines the anterior boundary between NC and forebrain by inhibiting canonical Wnt signaling [67]. In both mice and *Xenopus*, the Wnt antagonist Dkk1 is secreted by the prechordal mesoderm to inhibit NC formation and prevents the anterior neural fold from transforming into NC [68].

## 4. Wnt Signaling and Crosstalk with Other Signaling Pathways in NC Delamination and EMT

**Figure 4:**
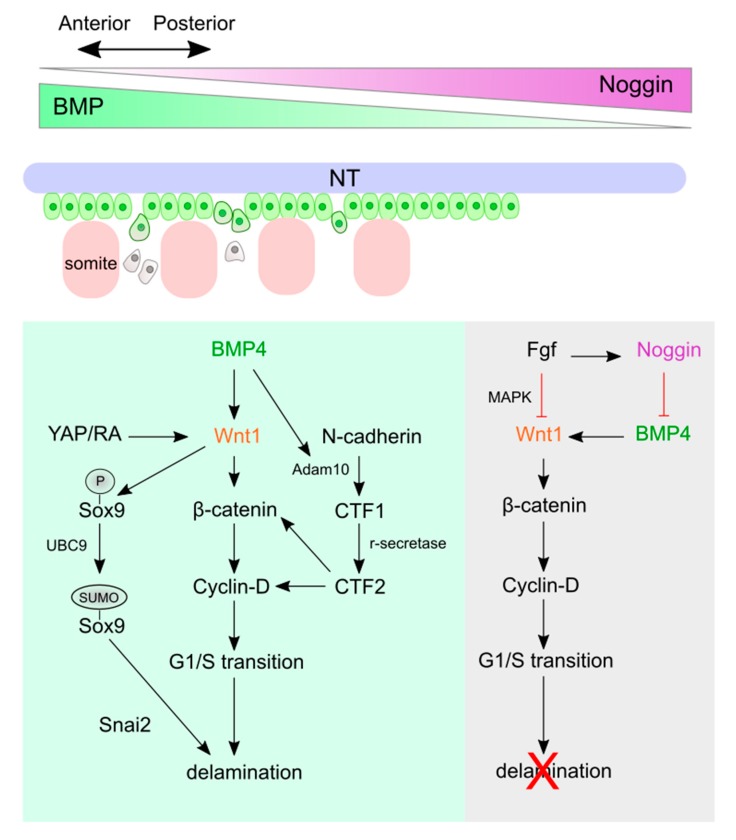
Bmp/Wnt signaling regulates trunk neural crest cell delamination. Undefined factors from somites inhibit *Noggin* (*purple*) expression anteriorly, creating a gradient Bmp (*green*) activity with a high level in anterior and low level in posterior of the dorsal neural tube. Bmp4, Yap, and RA signaling induce canonical Wnt1 expression, leading to Cyclin D1 transcription and G1/S transition to promote NC cell emigration. However, at the segmental plate mesoderm at the posterior region, Fgf signaling maintains high levels of Noggin that inhibits Bmp activity. Low Bmp activity blocks Wnt signaling, which prevents NC cell delamination from the caudal neural tube.

After induction and specification, NC cells emigrate from the dorsal neural tube by undergoing EMT. Although all NC cells undergo EMT and become migratory, differences between delamination of cranial NC cells and trunk NC cells exist [131]. For example, cranial NC cells delaminate from the neural tube together in mice and *Xenopus* [132,133,134]. However, trunk NC cells undergo EMT individually. Moreover, in chicks, the delamination and EMT of cranial NC cells are irrespective of the cell cycle, but almost all trunk NC cells delaminate in S phase [135]. Despite these axial level differences, Wnt signaling participates in the regulation of delamination for both cranial and trunk NC cells [136,137] (Figure 4; Table 2). In order to emigrate from the neural tube, NC cells need to firstly, have an appropriate substratum for migration; secondly, lose intercellular adhesion; and thirdly, obtain migratory ability [138]. Wnt interacts with Bmp, Fgf, retinoic acid (RA), and Yes-associated-protein (YAP) signaling in response to the extracellular microenvironment [92,139]. If the microenvironment is suited for NC migration, such as the segmental plate mesoderm, canonical Wnt signaling induces G1/S transition and prepares NC cells for EMT in the chicken embryo [92,137,139]. Several essential signaling molecules have been identified which regulate NC intercellular adhesion and motility, including the Rho family of small GTPases [140], cadherins [141], and the non-canonical Wnt/planar cell polarity (PCP) signaling [37]. The role of Wnt/PCP signaling in controlling polarized NC cells during migration will be discussed later. Here, we will focus on Wnt interactions with different signaling pathways to regulate NC delamination.

### 4.1. Wnt Signaling and Crosstalk in Trunk NC Delamination and EMT

Canonical Wnt signaling is required for the delamination of trunk NC cells, and its disruption blocks NC emigration in chicks [137,146]. Rabconnectin-3a (Rbc3a) is a v-ATPase-interacting protein expressed in pre-migratory NC cells of zebrafish and regulates Wnt signaling by controlling intracellular trafficking of Fzd7, while its depletion disrupts Wnt signaling and blocks NC cell emigration in zebrafish [147]. In chicken embryos, the signals from developing somites inhibit *Noggin* expression in the dorsal neural tube, resulting in high Bmp activity [137]. Bmp4, in turn, up-regulates its downstream target gene, *Wnt1* in chicks [137]. Through the canonical Wnt signaling pathway, Wnt1 positively regulates transcription of *cyclin D1* and G1/S transition of NC cells in chicks [137]. Since most trunk NC cells delaminate during the S phase in chicks, Wnt-mediated Bmp-dependent G1/S transition is required for NC cell delamination at the rostral segmental plate [135]. However, the segmental plate mesoderm expresses a high level of Noggin, which inhibits Bmp activity and expression of *Wnt1* and *cyclin D1*, blocking the emigration of NC cells from the caudal neural tube [137]. In chicks, Bmp/Wnt signaling also induces Sox9 phosphorylation. Phosphorylated Sox9 can be SUMOylated in vivo to facilitate interaction with Snai2 to trigger NC delamination [162].

N-cadherin belongs to the Ca^2+^-dependent cell adhesion family. It contains an intracellular β-catenin-binding domain, a transmembrane, and five extracellular cadherin-binding domains [167,168]. In quails, overexpression of N-cadherin inhibits NC delamination by reducing G1/S transition [143]. The cleaved β-catenin-binding cytoplasmic tail of N-cadherin, C-terminal fragment 2 (CTF2), could induce β-catenin and cyclin D1 transcription and therefore stimulate NC delamination [143]. Bmp4 regulates the cleavage of N-cadherin through Adam10. Adam10 cleaves N-cadherin into CTF1, which will be further cleaved by γ-secretase to form soluble CTF2 [143].

Rho signaling has also been shown to regulate NC delamination through Rock activity. In chicken embryos, *RhoA* and *RhoB* are expressed in the dorsal neural tube at stages corresponding to the delamination of NC cells [158]. Rho/Rock signaling is regulated by Bmp/Noggin and active at the membrane of NC cells before they undergo EMT [158]. The Rho/Rock signaling stabilizes N-cadherin at the cell membrane, inhibiting NC EMT. Upon delamination, Rho/Rock activities are downregulated, causing a loss of stress fibers and decreased N-cadherin-mediated adhesion [158]. Altogether, these studies suggest that Bmp/Wnt signaling regulates NC delamination through four separate yet converging pathways.

Other than Bmp, Wnt also interacts with YAP, Fgf, and RA signaling to regulate NC EMT. Fgf signaling blocks NC cell emigration by inhibiting the expression of *Wnt1* in premature chicken NC cells [92]. Fgf signaling also maintains the expression of *Noggin* in the caudal neural tube, whereas RA signaling induces the expression of *Wnt1* in the dorsal neural tube and controls the initiation of NC cell emigration. Therefore, Fgf and RA signaling play opposing roles to control the timing of NC EMT [92]. The Hippo signaling pathway controls many aspects of developmental processes, such as cell proliferation and survival. YAP, the main effector of Hippo signaling, interacts with a transcriptional co-activator called TAZ to regulate gene expression in response to specific molecular and mechanical signals from the microenvironment [169]. In chicken embryos, YAP regulates Bmp and Wnt signaling and stimulates G1/S transition, survival, and delamination of pre-migratory NC cells [139].

Although canonical Wnt signaling has been suggested to be required for NC delamination, other studies show that emigration of neural crest cells needs transient Wnt inhibition. Tracing the expression of a Wnt-responsive reporter in chicken embryos show that pre-migratory NC cells exhibit endogenous canonical Wnt activity. However, this activity will be transiently inhibited during chicken trunk NC and *Xenopus* cranial NC delamination [146]. Activation of the small intracellular scaffold proteins Dact1/2 is required for the inhibition of Wnt/β-catenin signaling for NC delamination [146]. Dact1/2 blocks canonical Wnt signaling by binding to β-catenin and accumulating it in nuclear bodies to prevent it. Therefore, Dact1/2 prevents β-catenin from interaction with TCF transcriptional co-activator and inhibits Wnt signaling [146]. *Dact2* is expressed in the trunk NC in chicken embryos, whereas pre-migratory NC expresses *Dact1* in *Xenopus* embryos [170]. A chicken neural tube explant assay showed that knockout of *Dact1/2* does not influence the motility of NC cells, but forces their release from the dorsal neural tube. These results seem conflicting but may indicate that Wnt signaling plays complicated roles during NC EMT. For example, NC cells must lose intercellular adhesion with neural epithelium and increase motility to delaminate, and Wnt signaling regulates cadherins involved in this adhesion [140].

In chicken NC, Wnt3a increases expression of cadherins *Cdh7* and *Cdh11*, which further accumulate at cell-cell interfaces [141]. In zebrafish, Rbc3a-deficient NC cells also display increased *Cdh11* expression levels during EMT [147]. Overexpression of *Cdh11* prevents NC migration, suggesting a requirement for Wnt inhibition for NC cells to lose intercellular adhesion and undergo EMT [124]. Additionally, as discussed before, Rho/Rock activity is repressed during EMT. However, activation of Rho by Wnt signaling has been suggested during *Xenopus* gastrulation [171]. Therefore, the role of canonical Wnt/β-catenin signaling in trunk NC delamination remains unclear.

### 4.2. Wnt Signaling and Crosstalk in Cranial NC Delamination and EMT

Unlike trunk NC, canonical Wnt signaling is repressed during delamination of cranial NC. *Draxin,* which is temporarily expressed in pre-migratory cranial NC cells before EMT, interacts with Lrp extracellularly, and inhibits canonical Wnt signaling in chicken embryos [136]. Draxin activity causes a decrease in *Snai2* expression and an increase in *Cad6b* expression to block the emigration of pre-migratory cranial NC cells. However, when cranial NC cells become migratory during EMT, *Draxin is* downregulated and canonical Wnt signaling is activated, which represses *Cad6b*, and cranial NC can delaminate from the neural tube. Therefore, Draxin controls the timing of cranial NC EMT [136]. In 1982, Newgreen and Gibbins suggested that physical barriers, such as the basal lamina, must be lost for NC cells to emigrate from the neural tube, but it was not experimentally confirmed for many years [138]. A recent study in chicken embryos finally found that during cranial NC EMT, the basement membrane protein laminin continually undergoes remodeling [148]. When pre-migratory NC cells are induced and localized at the dorsal neural tube, the basement membrane will form a space between the non-neural ectoderm and neural tube, which is termed “Regression” [148]. As cranial NC cells begin to delaminate, the basement membrane expands at the junction of the neural tube and ectoderm to encapsulate NC and creates a physical barrier that blocks NC delamination. This stage is called “Expansion” [148]. Finally, the lateral laminin barrier disappears and a laminin-lined “channel” forms, and cranial NC cells complete EMT and migrate through the “channel.” Draxin is involved in the channel formation [148]. *Draxin* knockdown inhibits basement membrane remodeling during the regression stage, and ectopic introduction of *Draxin* inhibits the dissolution of the lateral laminin barrier [148]. *Snai2* has been shown to rescue the formation defects of laminin channel from *Draxin* overexpression, suggesting that Wnt/β-catenin signaling is required for the formation of laminin channels and NC EMT at least in chicks [148].

## 5. Wnt Signaling in NC Migration

After induction and delamination, NC cells migrate long distances to contribute to the development of various tissues [172]. A common feature of all migrating NC cells is that they organize into discrete streams [54,173]. During migration, NC cells are influenced by a large number of activating and inhibitory signals [131,173]. Therefore, the direction of NC migration is regulated by many different signals present in the local environment, including chemotactic signaling, cell-cell interactions, and the extracellular matrix [131,172,173,174].

**Figure 5:**
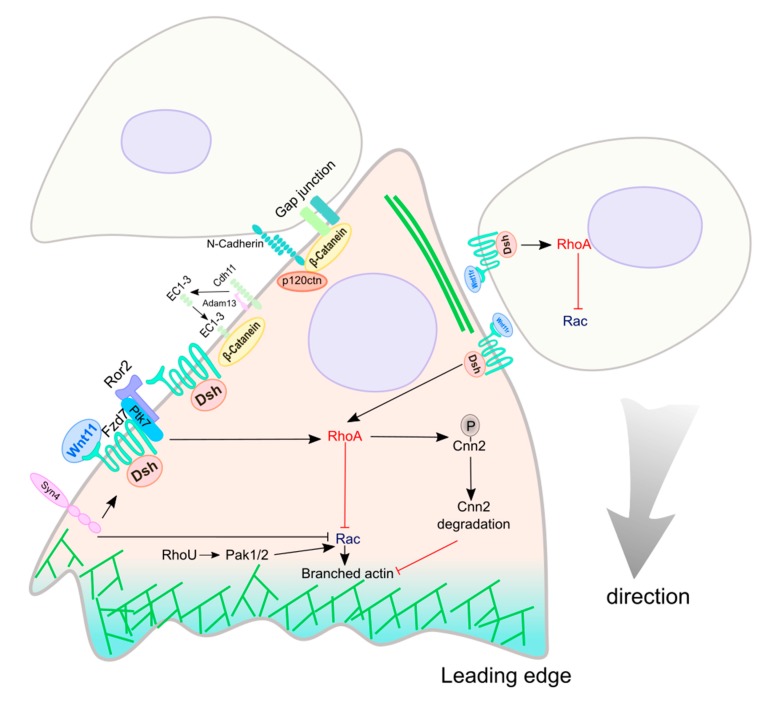
The role of Wnt signaling in CIL (contact inhibition of locomotion)-mediated directional migration of neural crest cells. Cell-cell interaction between NC cells localizes and activates Dsh (Dvl) at the cell membrane of the contact point, activating the small GTPase RhoA. The activation of RhoA is at least partly regulated by non-canonical Wnt signaling. RhoA inhibits Rac activity at the trailing edge of the cell, restricting a maximal Rac activation at the leading edge (*green*). Rac stimulates branched actin polymerization and drives the directed migration of NC cells.

NC cells need to be polarized to migrate directionally (Figure 5). Protrusions, such as filopodia and lamellipodia, are formed at the leading edge of migrating cells, while a retraction region is usually formed at the trailing edge [54]. Rho family GTPases have been shown to regulate this directional polarity by controlling the polymerization of actin at the leading edge [57]. Rac proteins regulate actin polymerization by activating actin nucleating proteins, such as the Arp2/3 complex [175]. Rho proteins activate Rock (Rho-associated kinase) and induce stress fibers [57]. During NC cell migration, environmental signals control the polarized localization of Rho GTPases and regulate the direction of NC cell migration. However, chemoattractants have been shown to be unable to establish the directionality of NC cell movement due to lack of in vivo chemoattractant gradients through the long migratory routes [172,176]. Therefore, movement directionality is more likely controlled by local signals. Cell-cell interactions such as contact inhibition of locomotion (CIL) have been shown to directly affect the localization of Rho GTPases and NC cell polarity [54,172]. CIL is a process by which a cell paralyzes protrusions in response to a collision with another cell to cease migration in that direction [177]. NC cells exhibit CIL both in vitro and in vivo, and physical contact between two NC cells inhibits their protrusions, leading to movement in opposite directions after collision [54,172,173,176]. At high density, only cells adjacent to a free region may migrate away from the cluster. Moreover, in vivo, CIL can cause the directional migration of NC cells since not all areas are available for migration [54,172,173]. Non-canonical Wnt signaling has been proposed to link CIL with the asymmetric distribution of Rho GTPases during NC migration [54].

### 5.1. Non-Canonical Wnt Signaling in NC Migration

Many studies have revealed the role of the non-canonical Wnt planar cell polarity (PCP) signaling pathway as the primary source of signals that direct NC cell migration (Figure 5; Table 2). Different factors involved in PCP signaling localize at the cell contact points during CIL. One example is the translocation of Dvl from cytoplasm to membrane during NC migration [178]. Different mechanisms control the localization of Dvl. In *Xenopus*, two non-canonical Wnt ligands, Wnt11 and Wnt11r, are required for cranial NC migration as extracellular signals. Before migration, *Wnt11r*, a *Xenopus* homolog of the mammalian *Wnt11* gene, is expressed medially while *Wnt11* is expressed lateral to the first migrating NC cells, which express the Wnt receptor *Fzd7* [37,166]. Disruption of Wnt11/Fzd7 signaling causes NC cells to generate fewer cell protrusions and lamellipodia at the leading edge during migration, suggesting Wnt11 controls NC migration through the PCP signaling pathway [37]. On the other hand, blocking Wnt11r causes cells to lose contact-mediated inhibition, suggesting that Wnt11r may act as a repellent signal that causes cranial NC cells to move away from the neural plate [166]. Moreover, Wnt11 or Wnt11r, Fzd7, and Dvl also accumulate at the contact site when migrating NC cells collide, suggesting a significant role of Wnt/PCP signaling in CIL and NC migration directionality [179].

Another protein that is essential for recruiting Dvl to the cell membrane is protein tyrosine kinase 7 (Ptk7). Ptk7 is a transmembrane pseudokinase which regulates the Wnt/PCP signaling pathway [180]. *Xenopus* Ptk7 is expressed in migratory NC cells and interacts with Ror2 through its extracellular domain [156]. Knockdown of *Ptk7* inhibits the motility of NC cells and results in a rounded rather than a disperse shape in vitro, while the kinase activity of Ror2 can rescue the Ptk7 loss of function phenotype [156]. These results suggest that the Ptk7/Ror2 complex regulates non-canonical Wnt signaling and NC migration. In *Xenopus*, Ptk7 forms a complex with Fzd7 and Dvl, suggesting Ptk7 is also required for Fzd7-mediated recruitment of Dvl to the cell membrane. Knockdown of *Ptk7* inhibits cranial NC migration, suggesting Ptk7 intersects with the PCP signaling pathway through Dvl localization to regulate NC mobility [157]. The accumulation of Dvl at the cell membrane leads to the localized activation of RhoA in *Xenopus* [179]. Activated RhoA antagonizes Rac, leading the retraction of protrusions at cell-cell contacts at the trailing edge of a migrating NC cell [57,175,179].

After cell-cell contacts cause the localized activation of Rho GTPases, Wnt/PCP signaling (Wnt11) activates the actin-binding protein calponin 2 (Cnn2) in both chicks and *Xenopus* [145]. In pre-migratory NC cells, Cnn2 is phosphorylated by RhoA/Rock signaling, leading to its degradation. However, in the early migratory NC cells, RhoA activity restricts Cnn2 to the leading edge of migrating NC cells, which causes the formation of a polarized cortical actin network [145].

Non-canonical Wnt signaling also activates the atypical RhoU GTPase during cranial NC cell migration [159,160]. The level of RhoU activity is essential for NC cells to form polarity and generate adhesive structures [160]. Explants from RhoU-depleted *Xenopus* embryos show a rounded phenotype and reduced adhesion to the substrate [160]. Overall, these experiments suggest that RhoU regulates the direction of cranial NC migration in *Xenopus*. Further studies show that RhoU controls cranial NC migration by regulating polarized cell adhesion [160]. Many effectors have been identified for RhoU such as PAKs (P21 activated kinases), including Pak1 and Pak2, which are known to participate in cell adhesion and motility [160,181]. PAK activates Rac1 and induces the formation of lamellipodia [175,182]. Therefore, RhoU regulates NC migration through the Pak1–Rac1 signaling pathway. The non-canonical Wnt4 also induces the expression of *Pescadillo* (*Pes1*) in *Xenopus* [155]. Pescadillo is a nuclear protein involved in ribosomal biogenesis and gene transcription [183,184]. Pescadillo knockdown blocks cranial NC migration and also triggers p53-mediated apoptosis, suggesting dual roles of Pescadillo in NC migration and survival [155].

Moreover, non-canonical Wnt signaling modulates the extracellular matrix to regulate the direction of NC migration in *Xenopus* and zebrafish. In migrating NC cells, the proteoglycan Syndecan-4 (Syn4) is activated by fibronectin. Activated Syn4 directly inhibits Rac activity [163]. Syn4 also interacts with the Wnt/PCP pathway through Dvl to inhibit Rac at the trailing edge of migrating NC cells, resulting in the formation of cell protrusions such as filopodia and lamellipodia at the leading edge [163]. Since persistent migration depends on the orientation of cell protrusions, Wnt/PCP and Syn4 signaling control the direction of NC migration.

Trunk NC cells migrate along three different pathways: a dorsolateral pathway [185,186], a ventro-lateral pathway [187], and a ventro-medial pathway [185,186]. In the ventro-lateral pathway and the ventro-medial pathway, the continuous migration sheet of NC cells rearranges into narrow, restricted streams. In zebrafish, the initiation of this segmentation is triggered by Nrp2/Sema3F signaling [153]. However, maintenance of the segmental migration is regulated by Wnt/PCP signaling [153]. The zebrafish Wnt11r binds to the surface of the adaxial muscle cells at the central portion of the somite. Wnt11r-Dvl signaling activates the expression of a muscle-specific receptor kinase (MuSK). MuSK signaling possibly modifies the extracellular matrix at the central region of the somite [153]. High-resolution imaging finds that NC cells interact with the extracellular microenvironment using their filopodia and guide the direction of their migration. Therefore, Wnt11r-MuSK signaling keeps NC cells into restricted streams in zebrafish embryos [153].

### 5.2. Canonical Wnt Signaling in NC Migration

The role of canonical Wnt signaling in controlling NC migration is less well understood. In *Xenopus*, canonical Wnt activity is decreased in migrating NC cells, while its activation inhibits NC migration [151]. Moreover, inhibition of Efhc1, a ciliary component, upregulates *Wnt8*, leading to defective NC migration in *Xenopus* [149]. However, in vitro studies show that although β-catenin is localized at the intercellular contact regions and associates with N-cadherin in most migrating NC cells, early migrating cells have β-catenin in their nuclei, suggesting that canonical Wnt signaling may be only transiently required in early migrating NC cells [150]. Recent studies in *Xenopus* also show that canonical Wnt signaling is activated in the cranial NC cells shortly after delamination, which drives the re-expression of *Snai2* in early migratory NC cells [188]. In zebrafish, Lrp5 is required for cranial NC migration. Knockdown of *Lrp5* leads to disrupted migration of cranial NC cells in the branchial stream [152]. In chicks, Wnt3a enhances NC migration along the distal medial pathway [165]. In vitro studies show that Wnt3a induces the expression of *Cdh11* [141]. Cdh11 is cleaved by Adam9 and Adam13 to generate a shed extracellular fragment of *Cdh11* (*EC1-3*). EC1-3 promotes *Xenopus* cranial NC migration through an unknown mechanism [189,190]. Interestingly, other than cleavage of Cdh11, Adam13 also regulates cranial NC cell migration by controlling the expression of many genes through its cytoplasmic domain (C13), such as the protease calpain 8 [142,191]. Gsk3 and Polo-like kinase (Plk) regulate the activity of Adam13 by successive phosphorylation of its C13 domain at two sites (first at S752 and S768 by Gsk3, second by Plk at T833). The phosphorylation of Adam13 is critical for cranial NC migration [142]. In *Xenopus*, *Fzd4* mRNA can be alternatively spliced to generate a secreted Frizzled related protein (Sfrp) Fz4-v1 [192]. Fz4-v1 regulates canonical Wnt signaling in a non-autonomous manner [161]. Knocking down *Fz4-v1* blocks NC cell migration [161]. The vestigial-like 3 (Vgll3) protein is expressed at hindbrain rhombomere 2 to activate the expression of *Wnt5a* and *Wnt8b* in *Xenopus* [164]. Depletion of Vgll3 causes a down-regulation of myosin X that is essential for cell-cell adhesion [193]. These results suggest that Vgll3 regulates NC migration through modification of their adhesion properties [164].

Canonical Wnt signaling is also involved in establishing transient cell-cell contacts during NC migration. This process possibly is regulated by localizing N-cadherin to filopodial tips of NC cells, which has been shown to be regulated by canonical Wnt signaling in zebrafish [154]. Wnt signaling activates the expression of *Ovo1,* a transcription factor gene [194]. Ovo1 inhibits the expression of several genes involved in intracellular trafficking, such as *rab12*, *rab11fip2*, *rab3c*, and *sec6*, which maintain the balance of cytoplasm and membrane-localized N-cadherin in zebrafish [154]. In mice, N-cadherin and β-catenin colocalize with the gap junction protein, connexin 43 or α1 connexin (Cx43α1) in migrating cardiac NC cells [144]. Further studies show that Wnt1 and N-cadherin regulate gap junction channels in NC cells, suggesting that cadherin-based adherents junction controls NC migration by modulating gap junction communication [144]. An Armadillo protein, p120 catenin (p120ctn), links gap junctions to the actin cytoskeleton [195]. N-cadherin and Cx43α1 interact with p120ctn and regulate cell motility through the Rho GTPases in mice [144].

## 6. Wnt in NC Multipotency and Fate Determination

The fact that the NC cells can generate many cell and tissue types makes them represent a multipotent stem cell population. Several studies have been performed in vivo to address the developmental potential of individual NC cells by retrovirus-mediated gene transfer or dye injection to label cell lineages [196,197,198]. These studies showed that at least some NC cells generate different cell types in vivo, including smooth muscle cells, neurons, glia, and melanocytes [198,199]. Cultured NC cells from mouse neural tube explants derived prior to NC cell migration demonstrated their multipotency in vitro [200]. After delamination from the neural tube, NC cells can differentiate into various derivatives. The multipotency of chicken pre-migratory NC cells has also been demonstrated using a clonal culture system [201]. A few studies proposed that pre-migratory NC cells are fate-determined before delamination in chicks [202,203]. A recent study employed advanced genetic fate mapping approaches to convincingly demonstrate that both pre-migratory and migratory trunk NC cells are multipotent in mice [204].

### 6.1. Wnt Signaling and Crosstalk in Maintaining NC Multipotency

In addition to demonstrating the stem cell nature of pre-migratory and migratory NC cells, in vitro studies have also revealed environmental signals regulating multipotential maintenance in NC cells [19]. Combinatorial canonical Wnt and Bmp proteins sustain persistent expression of the NC cell markers p75 and Sox10, suppress sensory neurogenesis and maintain multipotency in early NC stem cells [205] (Table 3). Further studies showed that Bmp/Wnt treatment induces the expression of a chromatin remodeler, chromodomain helicase DNA-binding protein 7 (Chd7), which maintains the multipotency of NC cells [27]. In mice, Chd7 is expressed in the undifferentiated migratory trunk neural crest cells, the dorsal root ganglia (DRG) and sciatic nerve, which normally contain neural crest stem cells [206,207,208]. Therefore, Bmp/Wnt signaling can maintain multipotency in cultured NC cells and increase the number of multipotent cells in DRG in vivo [27]. A more recent study showed that a high concentration of canonical Wnt ligand at the dorsal neural tube directly induces the expression of Lin28a in pre-migratory chicken NC cells [209]. Lin28a is an RNA-binding protein that binds to let7 pre-microRNA and blocks its maturation into let7 miRNA [210]. Therefore, Lin28a protects neural crest specifiers from inhibition by let7 and maintains the multipotency of pre-migratory NC [209]. As NC cells migrate away from the dorsal neural tube, expression of Lin28a decreases, leading to increased let7 activation, resulting in a loss of stem cell identity [209].

### 6.2. Wnt Signaling in NC Fate Specification and Differentiation

Wnt signaling is also involved in the fate specification and differentiation of NC cells [7] (Table 3). In zebrafish, activation of canonical Wnt signaling in pre-migratory NC cells promotes pigment cell formation, but inhibition of Wnt signaling promotes neuronal fates [214]. However, in mice, activation of Wnt/β-catenin signaling in pre-migratory NC cells promotes a sensory neuronal fate at the expense of all other NC derivatives [215]. Genetic ablation of β-catenin in pre-migratory NC cells using Wnt1-cre suppresses the formation of both melanocytes and dorsal root ganglia [40]. These contradictory results may be due to taxa-specific differentiation mechanisms, but nevertheless suggest that the role of canonical Wnt signaling in NC lineage specification needs to be addressed further. A recent study in mice shows that induced activation of β-catenin in migratory NC cells using Sox10-cre promotes the formation of melanoblasts and melanocytes, and the repression of other lineages [39]. However, β-catenin activation at later stages, such as glial progenitors or melanoblasts, does not produce similar effects [39]. These results imply a narrow time window in which canonical Wnt signaling controls migratory NC fate determination. It also suggests that NC cells maintain multipotency both before and shortly after delaminating from the dorsal neural tube.

Wnt signaling also affects NC differentiation at late stages in mice. For example, disruption of Axin2 enhances canonical Wnt signaling, increasing osteogenic potential and regeneration of NC-derived frontal bone in adult mice [211]. Conditional knockout of Bmp2 in mouse NC cells increases the expression of *Dkk1* in epithelium and reduces Wnt activity, leading to tooth mineralization defects [212]. NC-secreted Bmp4 genetically interacts with Msx1, repressing Osr2-dependent expression of Wnt antagonists Dkk2 and Sfrp2 in mouse tooth organogenesis [213,218]. Cranial NC cells generate cranial mesenchyme, which further develops into the forebrain meningeal progenitors [220]. Studies in mice show that canonical Wnt signaling is required to maintain the meningeal mesenchymal progenitors [216]. In ocular NC cells, canonical Wnt signaling maintains the expression of *Pitx2*, which is required for eye development [217]. Protein arginine methyltransferase 1 (Prmt1) negatively regulates canonical Wnt signaling [221,222,223,224]. Disruption of Prmt1 in NC cells causes cleft palate and craniofacial defects [219].

## 7. Wnt Signaling in NC-Derived Cancers

NC derivatives, including melanocytes and glial cells, can transform into cancers such as melanoma, neuroblastoma, and glioblastoma [225]. Indeed, WNT signaling and NC regulators are tightly associated with these types of cancers. Melanoma that develops from the pigment-producing melanocytes is the severest type of skin cancers. Among all skin cancers, melanoma is the most malignant and causes about 80% of all skin cancer deaths [226]. The development of melanoma often involves mutations in BRAF, CDKN2A, CCND1, INK4A, and MAPK signaling pathway components, many of which are also important for the development of neural crest-derived melanocytes [227]. Studies using melanoma cell lines discovered mutations in β-catenin, suggesting that canonical Wnt signaling may play an important role in this type of cancers [228]. Other studies have also detected β-catenin accumulation in the cytoplasm and nucleus of melanoma cells [229,230]. Moreover, expression of WNT proteins, including WNT2, 4, 5A, 7B, and 10B, have been found in melanoma cells or their local environment [231]. However, the sources and spatiotemporal expression patterns of these WNTs as well as the precise role of canonical signaling in melanoma remains unclear [232]. For example, melanomas with high β-catenin levels are correlated with a lower proliferative index [233], suggesting that β-catenin may inhibit melanoma. However, other studies showed that high β-catenin level correlates with enhanced melanoma metastasis [234].

Neuroblastoma is the commonest type of cancers in infants with the median age of 17 months at diagnosis, reflecting the embryonic origin of the disease [235,236,237]. It develops from the NC-derived sympathoadrenal system [238,239]. WNT signaling is implicated in onset and progression of neuroblastoma [240]. The oncogene *MYCN*, which is important for NC development, has been found to be upregulated in neuroblastoma cell lines or patient samples [241,242]. Xenograft experiments showed that the expression level of *WNT5A* is higher in human IGR-N-91 neuroblastoma cells than control grafted cells [243]. This is the first evidence for WNT signaling playing a role in human primary neuroblastoma. High WNT5A levels are associated with low-risk neuroblastoma [243]. Further studies showed that the core WNT/PCP signaling components PRICKLE1 and VANGL2 directly inhibit canonical WNT signaling in neuroblastoma cells [244]. High expression level of *PRICKLE1* and *VANGL2* correlates with low risk of neuroblastoma [244]. In contrast, transcriptome analyses show that high expression of *WNT3A* or *WNT5A* correlates with longer survival, while high *WNT3* expression level indicates higher risk of neuroblastoma [245]. These seemingly conflicting results suggest that the role of WNT signaling in neuroblastoma needs to be further addressed.

WNT signaling is also implicated in tumors of NC-derived endocrine cells, which include pheochromocytoma and paraganglioma [239,246]. The WNT signaling components *β-CATENIN*, *DVL3*, and *GSK3* are overexpressed in the *MAML3* fusion-positive subtype of these tumors and *CSDE1* somatic mutation. *WNT4*, *WNT5A*, and *WNT11* are also overexpressed after truncated *MAML3* activation [246]. These results suggest that overactivation of both canonical and non-canonical WNT signaling pathways may play important roles in these tumors.

Glioblastomas form from astrocytes and can occur in either the brain or spinal cord [247]. Key components of the WNT signaling pathway have been shown to be altered in glioblastoma. For example, *CTNNB1, LEF1, TCF7L2, MYC*, and *CCND1* are not expressed, while *APC* and *GSK3* are downregulated in glioblastoma [248]. Overexpression of *WNT5A* increases proliferation of glioblastoma cells in vitro, while knockdown inhibits proliferation and tumorigenicity [249]. Notably, the NC specifier Sox10 has been found to be upregulated in human low-grade gliomas and a mouse model of glioma [250].

NC and cancer cells possess similarities in several aspects [251,252]. During development, NC cells undergo EMT, emigrate from the dorsal neural tube, migrate through the embryo. This process is similar to the early stages of metastasis, when cancer cells disseminate from the original location [227]. At molecular and cellular level, the similarity of the development of NC cells and metastasis of cancer cells is more significant. Many signaling pathways, such as the Wnt signaling pathway, are shared by these two processes. For instance, WNT signaling also plays crucial roles in maintenance of cancer stem cells and metastasis [253,254], which is comparable to the roles of Wnt signaling in NC stemness and migration. Thus, NC cells represent an excellent model to study how developmental processes can be re-activated and usurped by cancer cells [255]. Studying the changes in cell-cell junctions, cell polarity, signaling, transcription factors, and the role of Wnt signaling at each step during NC development may provide insightful mechanisms of how cancer processes and hopefully trigger novel ideas for cancer treatments.

## 8. Conclusions

Since William His identified NC in 1868 [73], great advances have been made in understanding NC development for one and a half centuries. NC cells represent a group of migratory, multipotent stem cell population which are unique to vertebrate evolution. Therefore, NC cells serve as an excellent model for biologists to study the molecular and cellular mechanism of various developmental and evolutionary processes like morphogenetic induction, cell motility, and fate specification. Environmental cues and transcription factors have been shown to control the induction, delamination, migration, and differentiation of NC cells. Wnt signaling is a key driver for most NC developmental processes. Mutations in Wnt signaling components are involved in many diseases and cancers. However, important questions remain for the role of Wnt signaling in NC development and disease. For instance, does Wnt signaling play a role in NC evolution? Does Wnt signaling play a conserved role in human NC development? What are the roles of epigenetic factors, such as non-coding RNAs and DNA/RNA/protein methylation in Wnt signaling regulation of NC development? Whether and how environmental factors modulate Wnt signaling and thus may influence NC development or cause NC-derived disease and cancer? It is no doubt that studying Wnt signaling in NC development can lead to a better understanding of diseases, which hopefully will translate into practical therapies.

## Figures and Tables

**Table 1:** Experimental findings of Wnt signaling molecules, modulators, and effectors in vertebrate neural crest induction and specification. (cKO, conditional knockout; GOF, gain of function; KO, knockout; LOF, loss of function; MO; morpholino).

Molecule	Role in Wnt Signaling	Experimental Approach	Function	Region	Species	Phenotype	Reference
ADAM13	upregulates canonical Wnt signaling by cleaving class B Ephrins	MO	LOF	cranial	*Xenopus*	defective NC induction (Snai2, Sox9, Foxd3) and head cartilage	[75]
ADAM19	upregulates canonical Wnt signaling by stabilizing ADAM13	MO	LOF	cranial	*Xenopus*	defective NC induction (Snai2, Sox9, Foxd3) and head cartilage	[76]
Aldh1a2	delay Wnt3a and Wnt8a expression	KO	LOF	trunk	mouse	diminished NPB specification (Msx1, Pax3)	[77]
Apoc1	downstream effector of canonical Wnt signaling	MO	LOF	cranial	*Xenopus*	defective NPB induction (Msx1, Pax3, Zic1), eyes and head deformation	[78]
Awp1	stabilizes Ctnnb1	MO	LOF	cranial	*Xenopus*	defective NPB induction (Msx1, Pax3), pigmentation and craniofacial cartilage	[79]
Axud1	downstream effector of canonical Wnt signaling	MO, dominant-negative construct	LOF	trunk	chick	defective NC inductions (Foxd3, Sox9, Sox10, Ets1)	[80]
Cdh11	competitive binding with Ctnnb1 to repress Wnt/Ctnnb1 signaling	MO	LOF	cranial	*Xenopus*	increased Wnt/Ctnnb1 signaling and NC induction (Sox10, Ap2)	[81]
Ctnnb1	coactivator for Tcf/Lef1 transcription factor	RNA injection, MO	GOF; LOF	cranial	*Xenopus*	expanded (GOF) or diminished (LOF) NC induction (Snai2, Twist)	[82,83]
Daam1	mediator of non-canonical Wnt signaling and actin polymerization	MO, mutations	LOF	cranial	*Xenopus*	defective NC induction (Twist, Sox8, Snai2, Sox10)	[38]
Dkk1	antagonist of canonical Wnt signaling	blocking antibody, Dkk1-null mouse	LOF	cranial	*Xenopus*, mouse	NC generated in the anterior neural fold, expanded cranial cartilages	[68]
Dkk2	positive regulator of canonical Wnt signaling independent of Gsk3b	MO	LOF	cranial	*Xenopus*	defective NC induction (Snai2, Twist1, Sox10), reduced craniofacial cartilages	[34]
Dvl (Dsh)	canonical and non-canonical Wnt signaling	mutants (dd1, dd2)	LOF	cranial	*Xenopus*	repressed NC induction (Snai2)	[37]
	PCP signaling	PCP mutants: ΔN-Dsh, Dsh-DEP+	GOF, LOF	cranial	*Xenopus*	expanded (GOF) or decreased (LOF) NC induction (Foxd3, Sox8, Snai2)	[84]
Fgf8a	induce Wnt8 in the paraxial mesoderm	MO, RNA injection	LOF, GOF	cranial	*Xenopus*	defective (LOF) NPB induction (Pax3), induced (GOF) NC in anterior neural plate	[83]
Fzd3	receptor for Wnt1	RNA injection, MO	GOF, LOF	cranial	*Xenopus*	Induced (GOF) or diminished (LOF) NC induction (Snai, Twist)	[85]
Fzd7	receptor in canonical Wnt/Ctnnb1 signaling	MO	LOF	cranial	*Xenopus*	inhibited NC induction, loss of pigment cells	[86]
Gbx2	direct target of canonical Wnt signaling	MO, RNA injection	LOF, GOF	cranial	*Xenopus*	diminished (LOF) or rescued (GOF) NPB specifiers (Pax3, Msx1)	[87]
Gsk3b	phosphorylation and degradation of Ctnnb1	RNA Injections	GOF	cranial	*Xenopus*	increased NC induction (Krox20, Ap2, Snai2)	[88]
Hes3	inhibition of Wnt/Ctnnb1 signaling	expression constructs	GOF	cranial, trunk	*Xenopus*	blocked NC specifiers (Snai2, Sox10), supernumerary pigment cells	[35]
kctd15	attenuate canonical Wnt/Ctnnb1 signaling	MO, mRNA injection	LOF	cranial	zebrafish	defective NC induction (Sox10, Foxd3), increased pigmentation, loss of jaw elements	[89]
		expression constructs	GOF	cranial, trunk	zebrafish	increased NC induction (Foxd3, Sox10), loss of pigmentation, small head	[89]
Krm2	promotes Lrp6-mediated Wnt signaling in the absence of Dkks	MO, RNA microinjection	LOF, GOF	cranial	*Xenopus*	diminished (LOF) NC markers, ectopic NC-derived structures (GOF)	[90]
Lrp6	Wnt co-receptor	normal and mutant RNA injection	GOF, LOF	cranial	*Xenopus*	induced (GOF) or diminished (LOF) NC specifier Snai2	[47,90]
Mark2 (Par-1)	bind to Dvl and regulated by Wnt5a/Wnt11	MO, mRNA injection	LOF	cranial	*Xenopus*	repressed (LOF) or enhanced NC specification (Sox8, Foxd3, Snai2)	[84]
Rap2	stabilizes Lrp6 through TNIK kinase	MO, siRNA	LOF	cranial	*Xenopus*	abrogate ectopic expression of NC markers Snai, Foxd3	[91]
retinoic acid	regulate Wnt1 and Wnt3a expression	vitamin A deficiency	LOF	trunk	quail	defective NPB (Pax7) and NC induction (Snai2 and Sox9)	[92]
Rgs2	regulates Wnt-Ppard-Sox10 signaling cascade	MO, dominant negative rgs2 construct	LOF	cranial	zebrafish	increased NC induction (Sox10, Snail1b), reduced cranial cartilage formation	[93]
RhoV	downstream effector of canonical Wnt signaling	MO	LOF	cranial	*Xenopus*	defective NC induction (Sox9, Sox10, Snai), abnormal craniofacial skeletons	[94]
Ror2	co-receptor in non-canonical Wnt/PCP signaling	MO	LOF	cranial	*Xenopus*	defective NPB induction (Gbx2, Zic1, Msx1, Msx2), decreased BMP signaling at NPB	[84,95]
Skip	a potential scaffold in Ctnnb1/Tcf transcriptional regulation	MO, siRNA	LOF	cranial	*Xenopus*	defective NC induction (Snai2, Sox3, Foxd3), loss of pigment cells	[96]
Sp5	downstream effector of canonical Wnt and Fgf pathways	MO	LOF	cranial	*Xenopus*	defective NPB induction (Msx1, Pax3); defects in craniofacial cartilage and pigmentation	[97]
Tcf7l1	transcription factor	cKO by AP2α-Cre	LOF	cranial	mouse	anteriorly expanded NC specifiers (Foxd3, Sox9, Sox10, Pax3); exencephaly	[67]
		inhibitory mutants	LOF	cranial	*Xenopus*	defective NPB induction (Msx1)	[98]
		THVGR (hormone-inducible Tcf7l1)	GOF	cranial	*Xenopus*	increased NC induction (Snai2, Twist)	[82]
Wnt1	ligand, canonical pathway	dominant negative Wnt1	LOF	trunk	chick	repressed NC induction (Snai2)	[99]
		Wnt1 expressing cells	GOF	trunk	chick	inhibited NC induction	[100]
Wnt1/Wnt3a	ligand, canonical pathway	RNA and DNA Injections	GOF	cranial	*Xenopus*	increased NC induction (Krox20, Ap2, Snai2)	[88]
		Wnt1 and/or Wnt3a knockouts	LOF	cranial, trunk	mouse	defective NPB induction (Pax3), cranial skeletons, cranial ganglia	[101]
Wnt5a	ligand, non-canonical pathway	MO, dominant negative, RNA injection	LOF, GOF	cranial	*Xenopus*	defective (LOF) or enhanced (GOF) NC specification (Pax3, Foxd3, Sox8)	[84]
Wnt6	ligand, upstream of Dvl-Rho-JNK in PCP signaling	Wnt6 cell implantation, siRNA,	GOF, LOF	trunk	chick	induced (GOF) or diminished (LOF) NC induction	[100]
Wnt7b	ligand	RNA microinjection	GOF	cranial	*Xenopus*	increased NC induction (Snai2, Twist)	[102]
Wnt8	ligand, canonical pathway, downstream of Fgf8a	MO, mRNA injection	LOF, GOF	cranial	*Xenopus*	defective (LOF) NPB induction (Pax3, Sox8), rescued (GOF) NC in Fgf8a-deficient embryos	[83]
		dominant negative, RNA microinjection	LOF, GOF	cranial	*Xenopus*	defective (LOF) or increased (GOF) NC induction (Snai2)	[103]
Wnt11	ligand, non-canonical pathway	MO, dominant negative, RNA injection	LOF	cranial	*Xenopus*	defective (LOF) or enhanced (GOF) NC specification (Pax3, Foxd3, Sox8)	[84]
Wnt11r	ligand, non-canonical pathway	MO	LOF	cranial	*Xenopus*	defective NC specification (Foxd3, Sox8)	[84]

**Table 2:** Experimental findings of Wnt signaling molecules, modulators, and effectors in vertebrate neural crest delamination and migration. (cKO, conditional knockout; EMT, epithelial-mesenchymal transition; GOF, gain of function; KO, knockout; LOF, loss of function; MO; morpholino).

Molecule	Role in Wnt Signaling	Experimental Approach	Function	Region	Species	Phenotype	Reference
ADAM13	regulated by Gsk3 and Plk	MO	LOF	cranial	*Xenopus*	inhibited NC migration	[142]
Bmp4	stimulate Wnt1 expression	BMP4-coated microbeads	GOF	trunk	chick, quail	promoted G1/S transition and NC delamination	[137]
Cdh2	cleaved product CTF2 induces Ctnnb1 expression	expression vectors	GOF	trunk	quail	enhanced NC delamination	[143]
		KO	LOF	cardiac	mouse	elevated NC proliferation and reduced NC migration	[144]
Cnn2	downstream PCP signaling, actin dynamics	MO	LOF	cranial	chick, *Xenopus*	inhibited NC migration and reduced cartilage	[145]
Ctnnb1	coactivator for Tcf/Lef1 transcription factor	overexpression	GOF	trunk	chick	rescued NC delamination in Noggin-treated neural tubes	[143]
Dact1	repress Ctnnb1 as the transcriptional coactivator	MO, expression vectors	LOF, GOF	cranial	*Xenopus*	blocked (LOF) or enhanced (GOF) NC delamination	[146]
Dact2	repress Ctnnb1 as the transcriptional coactivator	RNAi, expression vectors	LOF, GOF	truck	chick	blocked (LOF) or enhanced (GOF) NC delamination	[146]
Dmxl2 (Rbc3a)	regulate Fzd7 endocytosis and enhance Wnt signaling	MO	LOF	cranial, trunk	zebrafish	defective NC migration, cardiac edema, reduced melanocytes	[147]
Draxin	repress Wnt signaling via Lrp5, modulate laminin	MO, CRISPR; expression vectors	LOF, GOF	cranial	chick	premature NC delamination (LOF), inhibited EMT (GOF)	[136,148]
Dvl (Dsh)	PCP signaling	PCP mutants (Dsh-DN, Dsh-DEP+)	LOF	cranial	*Xenopus*	repressed NC migration	[37]
Efhc1 (Efhc1b)	downregulate Wnt8a	MO	LOF	cranial	*Xenopus*	upregulated Wnt signaling and defective NC migration	[149]
Fgf8/4	inhibit Wnt1 expression	Fgf8/4-soaked beads	GOF	trunk	chick	repressed NC emigration or delamination	[92]
Fgfr1	inhibit Wnt1 expression	dominant-negative, inhibitor	LOF	trunk	chick	premature NC emigration	[92]
Gsk3	phosphorylation and degradation of Ctnnb1	Gsk3 inhibitors LiCl, BIO	Wnt GOF	trunk, cranial	chick, *Xenopus*	inhibited NC delamination and migration	[150,151]
Lef1	transcription factor, canonical pathway	inducible Lef1-GR	GOF	cranial	*Xenopus*	repressed NC migration	[151]
Lrp5	co-receptor	MO, CRISPR	LOF	cranial	zebrafish	defective NC migration, cranial skeleton malformation	[152]
Musk	downstream of non-canonical Wnt11r-Dsh signaling	transgenic fish, KO mouse	LOF	trunk	zebrafish, mouse	defective segmental NC migration	[153]
Noggin	inhibit Wnt1 expression	CHO-Noggin cells	LOF	trunk	Chick	inhibited G1/S transition and NC delamination	[143]
Ovo1	Wnt target, regulate intracellular trafficking of Cdh2	MO	LOF	cranial	zebrafish	defective migration of NC-derived pigment precursors	[154]
Pes1 (Pescadillo)	downstream of Wnt4/Fzd3	MO	LOF	cranial	*Xenopus*	increased apoptosis; defective NC migration, eye and craniofacial cartilage	[155]
Ptk7	co-receptor, interact with Ror2 in Wnt/PCP signaling	MO	LOF	cranial	*Xenopus*	defective NC migration	[156]
	required for Fzd7-mediated Dsh localization	MO	LOF	cranial	*Xenopus*	defective NC migration	[157]
Rara	regulate Wnt1/Wnt3a expression	dominant negative/active RA receptors	LOF, GOF	trunk	chick	diminished (LOF) or enhanced (GOF) NC emigration	[92]
RhoA, RhoB	regulated by Wnt6 [100]	dominant-negative, inhibitor, activator	LOF, GOF	trunk	chick	enhanced (LOF) or repressed (GOF) NC EMT/delamination	[158]
RhoU	activated by Wnt1 [159]	mutant construct, MO, RNA injection	LOF, GOF	cranial	chick, *Xenopus*	blocked (LOF) or impaired NC migration, reduced cartilages	[160]
Ror2	co-receptor, interact with Ptk in Wnt/PCP signaling	expression vector	GOF	cranial	*Xenopus*	rescued migration defect in Ptk7-deficient NC cells	[156]
Sfrp (Fz4-v1)	secreted splice variant of fz4 receptor	MO, mRNA injection	LOF, GOF	cranial, trunk	*Xenopus*	defective NC migration (LOF), altered Wnt signaling	[161]
Sox9	phosphorylated by Wnt1 and Bmp signaling	MO; point mutations	LOF	trunk	chick	failed to initiate NC delamination	[162]
Syn4 (Syndecan4)	interact with Wnt5/Dvl/PCP signaling	MO	LOF	trunk	*Xenopus*, zebrafish	diminished NC migration, reduced cartilage and melanocytes	[163]
Tcf7l1 (Tcf3)	transcription factor, canonical pathway	inducible Tcf3-VP16-GR, Tcf3ΔC-GR	GOF, LOF	cranial	*Xenopus*	impaired NC migration	[151]
Vgll3	induce Wnt5a and Wnt8b expression	MO, mRNA injection	LOF, GOF	cranial	*Xenopus*	impaired NC migration, trigeminal and profundal placodes	[164]
Wnt1	ligand, canonical pathway	Wnt1 producing cells	GOF	trunk	chick	inhibited NC delamination and migration	[150]
		Wnt1 DNA electroporation	GOF	trunk	chick	enhanced NC delamination	[143]
Wnt3a	ligand, canonical pathway	Wnt3a cells, melanoma cells	GOF	trunk	chick, human cell line	enhanced EMT and NC migration	[165]
Wnt5	ligand, PCP signaling	MO	LOF	trunk	*Xenopus*	defective NC migration	[163]
Wnt11	ligand, PCP signaling	expression vectors	LOF, GOF	cranial	*Xenopus*	inhibited NC migration	[37]
Wnt11r	ligand	MO, mRNA injection	LOF, GOF	cranial	*Xenopus*	repressed or rescued NC migrating	[166]
Yap (Yap1)	bidirectional crosstalk with Wnt and Bmp signaling	expression vectors, mutants, siRNA	GOF, LOF	trunk	chick, quail	stimulated (GOF) or inhibited (LOF) NC EMT and emigration	[139]

**Table 3:** Experimental findings of Wnt signaling molecules, modulators, and effectors in vertebrate neural crest proliferation and differentiation. (ca, conditional active; cKO, conditional knockout; GOF, gain of function; KO, knockout; LOF, loss of function; MO; morpholino).

Molecule	Role in Wnt Signaling	Experimental Approach	Function	Region	Species	Phenotype	Reference
Axin2	scaffold protein for Ctnnb1 degradation	KO	LOF	cranial	mouse	enhanced osteogenic potential and regeneration of NC-derived frontal bone	[211]
Bmp2	crosstalk with Wnt/Ctnnb1 signaling	protein to NC culture	GOF	truck	mouse	suppressed sensory neurogenesis of early NC stem cells	[205]
	repress Wnt antagonists Dkk1 and Sost	cKO by Wnt1-Cre	LOF	tooth	mouse	early tooth mineralization defects	[212]
Bmp4	repress Wnt antagonists Dkk2 and Sfrp2	cKO by Wnt1-Cre	LOF	tooth	mouse	bud-stage arrest of the mandibular molar tooth germs	[213]
Chd7	chromatin remodeler, activated by Wnt/Bmp	siRNA, DN, WT expression	LOF, GOF	trunk, DRG	mouse	inhibited (LOF) or maintained (GOF) undifferentiated state (Sox10, p75) of NCSC	[27]
Ctnnb1	coactivator for Tcf/Lef1 transcription factor	mRNA injection	GOF	cranial	zebrafish	promoted pigment and cartilage fates from NC cells	[214]
		ca by Wnt1-Cre in premigratory NC cells	GOF	trunk	mouse	suppressed melanocyte (Dct, Mitf) differentiation from premigratory NC cells	[39]
		ca by Sox10-Cre in migratory NC cells	GOF	trunk	mouse	ectopic melanocytes, inhibited other lineages from migratory NC cells	[39]
		cKO by Wnt1-cre	LOF	trunk	mouse	lack melanocytes and dorsal root ganglia	[40]
		ca, cKO by Wnt1-Cre	GOF, LOF	trunk, cranial	mouse	promoted (GOF) or blocked (LOF) sensory neurogenesis of NC stem cells	[215]
		cKO by PdgfraCreErt2, Dermo1Cre	LOF	cranial	mouse	forebrain meningeal hypoplasia derived from NC cells	[216]
		Wnt1-Cre	LOF	cranial	mouse	defective maintenance of Pitx2 expression in NC cells and abnormal eyes	[217]
		Wnt1-cre	LOF	cranial	mouse	affected NC survival and differentiation; failure of craniofacial development	[115]
let-7 miRNA	repressed by Wnt/Lin28a	electroporation of let-7 mimic	GOF	trunk	chick	down-regulation of NC multipotency, promoted differentiation	[209]
Lin28a	activated by Wnt	siRNA; MO; CRISPR	LOF	trunk	chick	suppressed NC multipotency (Sox10, Foxd3)	[209]
Msx1	repress Dkk2 and Sfrp2, interact Bmp4	KO	LOF	tooth	mouse	bud-stage arrest of the mandibular molar tooth germs	[213]
Osr2	upregulate Dkk2 and Sfrp2 expression	KO	LOF	tooth	mouse	supernumerary teeth	[213,218]
Prmt1	inhibit Wnt, Bmp and other signaling	cKO by Wnt1-Cre	LOF	cranial	mouse	decreased mesenchymal proliferation, cleft palate, craniofacial anomalies	[219]
Tcf7l1 (Tcf3)	transcription factor of Wnt/Ctnnb1 signaling	mutant mRNA injection	LOF	cranial	zebrafish	promoted neural fates, repressed pigment cells	[214]
Wnt1	ligand, Ctnnb1-dependent	Wnt1-expressing fibroblasts	GOF	trunk	mouse	promoted sensory neurogenesis of early NC stem cells	[215]
Wnt1 and Bmp2	ligands	Wnt1 cell and Bmp2 protein	GOF	truck	mouse	repressed neurogenesis and maintained multipotency of NC stem cells	[205]
Wnt3a and Bmp2	ligands	proteins to NC culture	GOF	trunk, DRG	mouse	maintained multipotency (Sox10 or p75) of NC stem cells	[27]
Wnt inhibitors	stabilize or elevate Axin	XAV939, IWR1	LOF	cranial	*Xenopus*	repressed NC differentiation and defective cartilage	[188]
